# Active surveillance in papillary thyroid carcinoma: not easily accepted but possible in Latin America

**DOI:** 10.20945/2359-3997000000168

**Published:** 2019-08-28

**Authors:** Anabella Smulever, Fabián Pitoia

**Affiliations:** 1 Universidad de Buenos Aires Division of Endocrinology Hospital de Clinicas University of Buenos Aires Buenos Aires Argentina Division of Endocrinology, Hospital de Clinicas, University of Buenos Aires Buenos Aires, Argentina

**Keywords:** Thyroid cancer, observation, active surveillance, low-risk

## Abstract

**Objectives:**

To determine the percentage of patients with papillary thyroid carcinoma (PTC) who accepted active surveillance as an alternative to surgery in our clinical practice and to describe the clinical characteristics and outcomes of patients with Bethesda category V and VI thyroid nodules who chose active surveillance.

**Subjects and methods:**

We included 136 PTC patients from the *Hospital de Clínicas*, University of Buenos Aires without (i) US extrathyroidal extension, (ii) tumors adjacent to the recurrent laryngeal nerve or trachea, and/or (iii) US regional lymph-node metastasis or clinical distant metastasis. PTC progression was defined as the presence of i) a tumor larger than ≥ 3 mm, ii) novel appearance of lymph-node metastasis, and iii) serum thyroglobulin doubling time in less than one year. For patients with these features, surgery was recommended.

**Results:**

Only 34 (25%) of 136 patients eligible for active surveillance accepted this approach, and around 10% of those who accepted abandoned it due to anxiety. The frequency of patients with tumor enlargement was 17% after a median of 4.6 years of follow-up without any evidence of nodal or distant metastases. Ten patients who underwent surgical treatment after a median time of 4 years of active surveillance (AS) had no evidence of disease after a median of 3.8 years of follow-up after surgery.

**Conclusion:**

Although not easily accepted in our cohort of patients, AS would be safe and easily applicable in experienced centers.

## INTRODUCTION

Papillary thyroid carcinoma (PTC) is the most common endocrine malignancy, accounting for about 1% of all cancers (1). An exponential increase in its incidence has been demonstrated over the last decade (2). However more than 50% of this increase is linked to the identification of intrathyroidal papillary microcarcinomas (PMCs) (3). The origin of this upward trend in the incidence of PMCs has not been elucidated, possibly due to the wider use of diagnostic imaging technology combined with greater access to health care and patients’ improved socioeconomic conditions (4,5). Most PTCs are non-palpable, and their diagnosis arises from ultrasonographic incidental findings or from the anatomopathological study of removed thyroid glands due to benign pathology (3,6). If never diagnosed and treated, most of these PMCs would remain stable without influencing overall survival, as shown in several autopsy studies, which revealed a prevalence of PMCs ranging from 4.2 to 35.6% (7-9). On the other hand, several authors warned about the medical costs of thyroid cancer treatment, which might expand to US$ 3.5 billion by 2030 in the United States (10).

At the moment, the therapeutic approach for patients with differentiated thyroid carcinoma should be individualized to differentiate patients who will benefit from a more aggressive therapy from those who may require a conservative approach (11). In this last group of patients, observation instead of immediate surgery emerges as a valid option.

Akira Miyauchi was the first investigator to propose active surveillance for patients with PMC, based on the fact that a small minority of these PMCs might progress (12). This researcher hypothesized that most PMCs remain latent without progression or with very slow progression, so it would not be harmful to delay surgery until diagnosis of significant size enlargement or development of nodal metastasis (13). This active surveillance strategy entailed performing neck ultrasound examinations every 6 to 12 months, and progression was defined as an increase in tumor diameter of 3 mm or more and/or the identification of lymph node/distant metastasis during the follow-up (12).

The first active surveillance study of PMCs was published in 2003, and it showed that tumor size remained stable or decreased compared to the baseline in more than 70% of the patients (12). The same and other researchers later confirmed these data (14-21). Additionally, Tuttle and cols. observed similar results in tumors up to 1.5 cm in size, a situation that supports active surveillance as a valid follow-up alternative (14).

However, this alternative’s applicability in patients with a diagnosis of PMCs needs to be evaluated considering the various scenarios, which usually include i) a medical team likely to follow this new approach, ii) patients who accept this strategy, and iii) a radiologist who knows what must be considered from each image study during the follow-up.

Due to the absence of any published experience in Latin America, the aim of this study was i) to determine the percentage of patients with PTC who accepted active surveillance as an alternative to surgery in our clinical practice and ii) to describe the clinical characteristics and outcomes of patients with Bethesda category V and VI thyroid nodules who underwent active surveillance at the Hospital de Clínicas at the University of Buenos Aires.

## SUBJECTS AND METHODS

We evaluated 136 patients with a diagnosis of PCMs eligible for active surveillance who attended our hospital before the indication of a surgical treatment.

Inclusion criteria were i) the presence of a single thyroid nodule classified as PTC (Bethesda category VI) or suspicious for PTC (Bethesda category V) (22); ii) tumor size of 1.5 cm or less in maximal diameter at diagnosis; iii) no clinical or radiographic evidence of extrathyroidal extension, invasion of local structures, or regional (N1) or distant metastases (M1); and iv) undetectable serum calcitonin levels.

Patients who did not meet the previously described criteria but had a high surgical risk or refused to be operated on were also included.

### Follow-up

Patients’ follow-ups included thyroid and neck ultrasonography examinations and assessment of serum TSH and thyroglobulin (Tg) and anti-thyroglobulin antibodies (TgAb) levels every 6 months. All patients had to follow-up for at least 6 months.

Patients with posterior tumors near the trachea or esophagus or located along the course of the recurrent laryngeal nerves or carotid were excluded.

Prospective active surveillance was considered when diagnosis of PTC was made at the beginning of follow-up and retrospectively when cytological diagnosis of Bethesda categories V and VI was established after surveillance of a single thyroid nodule that grew since a previous ultrasonographic follow-up.

Serum Tg levels were measured by an electrochemiluminescent method (ECLIA), Elecsys 2010 (Roche) with an analytical sensitivity of 0.04 ng/mL and functional sensitivity of 0.1 ng/mL. TgAb levels were measured by an electrochemiluminescent method, Elecsys Anti-Tg (Roche); values > 20 IU/mL were considered positive. Serum TSH was measured with a commercialized chemiluminescence assay kit (Siemens Advia Centaur^®^ XPT TSH3-UL) (reference range of 0.35 to 5.5 mU/l).

Additionally, the same operator performed a neck ultrasound with a linear 11 MHz transducer every six months.

### Indication of surgery

Surgery was recommended if i) the primary tumor grew 3 mm or more in the greatest dimension from the baseline in less than one year, ii) the greater diameter was larger than 1.5 cm during any moment of the follow-up, ii) serum Tg doubling time less than one year, or iii) evidence emerged of extrathyroidal extension, nodal or distant metastasis.

### Evaluation after surgery

Each patient was stratified after surgery with the modified 2015 ATA risk stratification system (low, intermediate, or high risk of recurrence) (23), which identified the mortality risk according to the eighth edition of the AJCC/UICC staging system (TNM Stage) (24). Patients were re-stratified according to their responses to therapy assessment compared to the previously published definitions (excellent response, indeterminate response, biochemical incomplete response, or structural incomplete response to therapy) (23,25).

Our hospital’s Ethical Committee approved the present study. All the participants provided written informed consent for data collection and tumor acquisition.

### Statistical analysis

Continuous data were presented as means (SDs) or medians (ranges) and interquartile range, as appropriate for each variable. Percentage change in size was calculated relative to the baseline estimated in ultrasound at diagnosis. A meaningful change in tumor size was defined as an increase greater than 3 mm from baseline before the initial fine-needle aspiration. The values obtained were compared using Chi^2^ for categorical variables and a t test for comparison of two means for continuous variables with normal distribution. The statistical tests used to compare the differences between the groups were the Mann-Whitney U test for skewed variables and the Wilcoxon signed-rank test for paired skewed variables. All statistical analyses were conducted using SPSS, 24.0 version (IBM Corp).

## RESULTS

Of 136 eligible patients offered the alternative of active surveillance, only 34 (25%) decided to undergo this approach. All patients who chose immediate surgery expressed anxiety about disease progression and uncertainty about identification of regional or distant metastases with continued observation. Therefore, a total of 34 patients with PTC smaller than 1.5 cm in diameter (Bethesda category VI) or suspicious for PTC (Bethesda category V) were included in the study. The mean follow-up was 48.8 ± 29.6 months (median, 42 months [range, 7-120 months]). Active surveillance was adopted due to i) the patient´s election to be monitored with observational management (n = 31; 91%), ii) high surgical risk (n = 2; 6%), and iii) surgical issues that needed to be addressed prior to the thyroid surgery (n = 1, 3%).

Baseline characteristics of patients included in the study are summarized in Table 1.

**Table 1:** Baseline characteristics of included patients

Variable	Patients with stable tumors (n = 28)	Patients with tumor increased (> 3 mm) (n = 6)
**Age at diagnosis, y**		
Median (range)	41 (15-79)	42 (26-79.7)
<60 y.o. (n)	30 (88)	5 (83)
**Sex, n (%)**		
Female	29 (85)	6 (100)
Male	5 (15)	0
**Citology, n (%)**		
Bethesda category V	10 (30)	1 (17)
Bethesda category VI	24 (70)	5 (83)
**Surveillance modality (%)**		
Prospective	24 (70)	4 (67%)
Retrospective	10 (30)	2 (33%)
**Extent of active surveillance (months)**		
Median (range)	42 (7-120)	41,8 (24-84)
**Tumor size (%)** ≤ 1 cm 1.1-1.5 cm	24 (70) 10 (30)	1 (17) 5 (83)
**History of autoimmune thyroid disease, n (%)**		
None	19 (56)	5 (83)
Autoimmune thyroiditis	13 (38)	1 (17)
Subacute thyroiditis	1 (3)	0
Graves’ disease	1 (3)	0
**TSH levels (mUI/mL)**		
Median (range)	0.63 (0.52-1.5)	1.03 (0.8-1.2)
**Levothyroxine treatment (%)**		
Yes	36	0
No	64	100

SD: standard deviation; n: number of patients; TSH: thyrotrophin.

**Figure 1:**
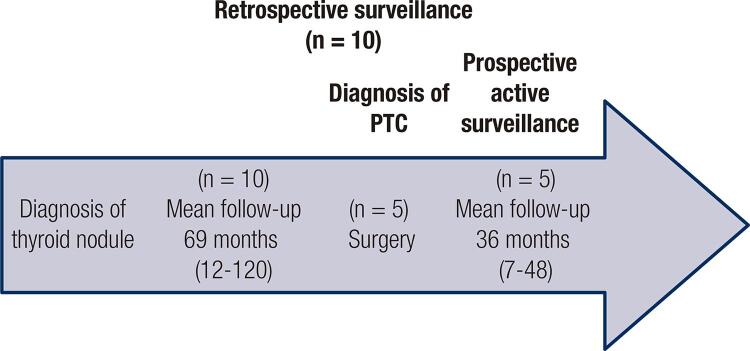
Graphical explanation of the concept of retrospective surveillance.

Active surveillance was prospective in 70% (n = 24) of patients with cytological diagnosis at the beginning of follow-up, and in the remaining 30% (n = 10), active surveillance was initially performed retrospectively, with the diagnosis of a PTC by FNAB after a mean of 69.9 ± 34.15 months of follow-up of a solitary thyroid nodule by neck ultrasonography. In this group, 50% (n = 5/10) of patients underwent surgery at the moment of inclusion, and the other 50% continued with a prospective active surveillance (n = 5/10) (Figure 1).

PTC: papillary thyroid carcinoma.

**Figure 2:**
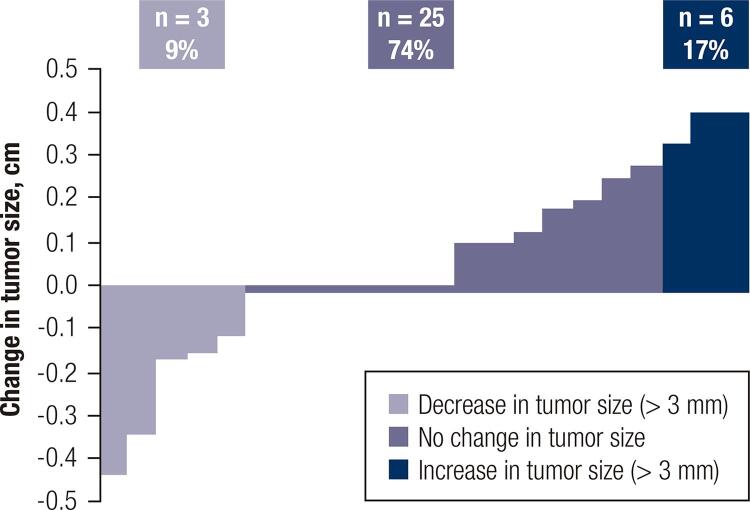
Percentage of change in tumor size (more or less than 3 mm) during active surveillance for each patient.

The median tumor size of the whole cohort was 9.20 mm ± 2.95 mm at diagnosis and 9.66 mm ± 3.10 mm at the end of follow-up or at the moment of surgery (p = NS). Tumor diameter growth of 3 mm or more was observed in 17% (n = 6), and 74% (n = 25) did not exhibit significant changes during follow-up. Additionally, 9% (n = 3) of patients showed a decrease in size of more than 3 mm (Figure 2).

It was not possible to identify a differential feature that was linked to tumor progression between groups. Although the majority of tumors that increased in size were larger than 1 cm, reliable conclusions about this trend cannot be obtained given the small number of patients who experienced tumor growth. The latter group’s characteristics are summarized in Table 1.

Mean and median serum Tg baseline levels were 34.84 ± 80.41 ng/ml and 7.5 ng/ml (0.2-320 ng/mL), respectively. Tg levels greater than 100 ng/ml were observed in two patients (5%) (106 ng/mL and 172 ng/mL, respectively), and only one patient showed a doubling time shorter than one year during follow-up.

At the time of writing this manuscript, four patients who experienced tumor growth were still under active surveillance, with no clinical or radiological evidence of extrathyroidal extension, lymph node, or distant metastases.

The clinical outcomes of the 102 patients who refused active surveillance can be observed in Table 2.

**Table 2:** Clinical outcomes of 102 patients who refused to undergo active surveillance and received surgery* during the first three months after the diagnosis of a papillary thyroid microcarcinoma

	Initial response	Response at the end of follow-up (median 32.6 months [12-56])			

		NED	IR	BIR	SIR
**Total (n = 102/136)**	**ER (n = 69,68%)**	98,5% (n = 68)	1,5% (n = 1)	–	–
	**IR (n = 29, 28%)**	55,1% (n = 16)	44,9% (n = 13)		
	**BIR (n = 1, 2%)**	–	–	100% (n = 1)	–
	**SIR (n = 1, 2%)**	50 % (n = 1)	–	–	50 % (n = 1)

* Total thyroidectomy in 80 patients and hemithyroidectomy in 22 patients.

ER: excellent response; IR: indeterminate response; BIR: biochemical incomplete response; SIR: structural incomplete response; NED: no evidence of disease.

### Outcomes in patients who switched from active surveillance to surgery

Surgery was performed on 29% (n = 10) of patients. In this group, 50% (n = 5) decided to undergo surgery during the prospective observation, and in the other half (n = 5), the indication of surgery was given at the moment of study inclusion due to the presence of a tumor with a diameter near 15 mm in a retrospective surveillance background, as previously explained. In the latter scenario, a patient with a single 15-mm nodule was included, in which the diagnosis of PTC was obtained after 108 months of follow-up of a thyroid nodule with negative FNAB result previously performed.

**Figure 3:**
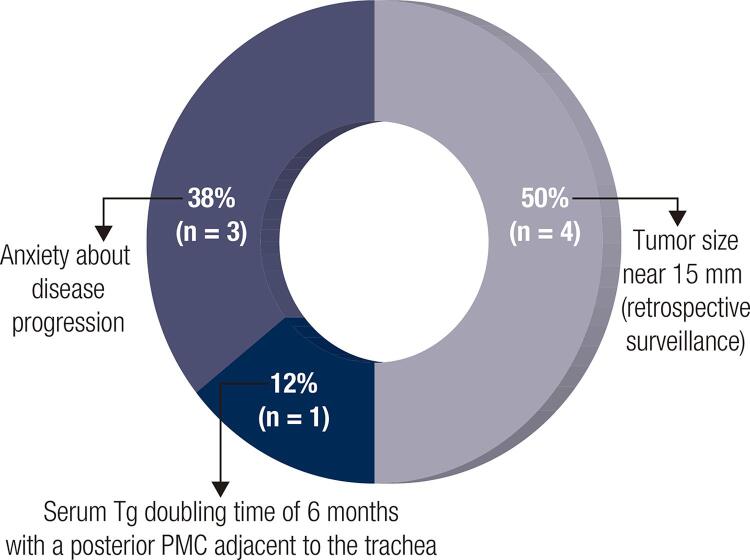
Causes of surgery despite no increase in tumor size

**Figure 4:**
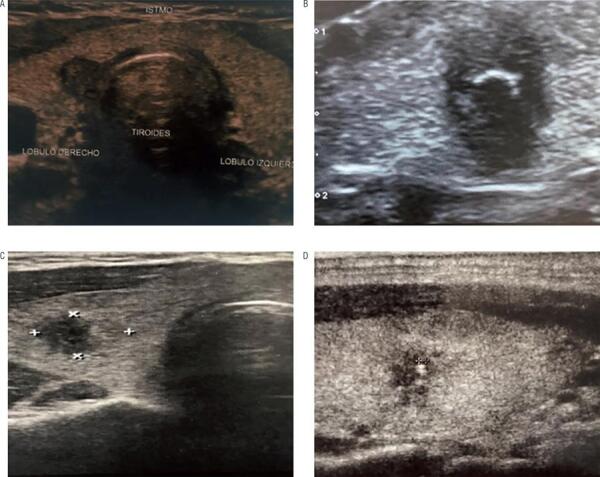
Ultrasonographic images of selected patients with PTC undergoing active surveillance.

Only two patients underwent surgery due to an increase in tumor diameter of 3 mm or more in the greatest dimension over baseline. Eight patients decided to undergo surgery despite no increase in tumor size. Among them, 3 patients returned to the clinic having undergone thyroidectomy for anxiety-related issues, representing 9% of the total cohort. For one additional patient, surgery was indicated after the observation of a doubling of serum Tg level in six months (from 172 ng/mL to 320 ng/mL) associated with a posterior tumor localization adjacent to the trachea, for whom active surveillance was previously agreed upon due to a high surgical risk (Figures 3 and 4).

**(A)** Eleven mm PTC with posterior localization adjacent to the trachea and duplication of Tg serum levels in less than one year. Active surveillance was previously agreed due to a high surgical risk. The patient underwent surgery after 12 months of follow-up with a low risk of recurrence tumor and an excellent response to treatment after 32 months of follow-up; **(B-D)** Markedly hypoechoic thyroid nodules with macro and microcalcifications and irregular margins (Bethesda category VI). Tumor size remained stable under active surveillance (12, 5 and 12 mm respectively) after 84, 30 and 53 months, respectively.

The majority of patients who decided to undergo surgery preferred total thyroidectomy even though lobectomy was offered as the ideal approach.

Ninety percent of the patients who received surgery were women, and the mean age at diagnosis of the PTC was 41.27 ± 11.9 years old. The median tumor size was 11.6 ± 2.69 mm at the beginning of active surveillance and 11.7 ± 2.72 mm before the surgical procedure (p = NS) after a mean follow-up of 56.2 ± 38.2 months (median, 45 months [range, 12-120 months]). All of these patients were classified as harboring a low risk of recurrence and TNM stage I. Seven patients (70%) had an excellent initial response to treatment, and one showed of the following responses: biochemical incomplete, indeterminate, and structural incomplete (representing 10% each).

The patient who presented a structural incomplete response to treatment received a central neck compartment dissection one year after total thyroidectomy. No patient showed evidence of disease at the end of follow-up (median, 46.4 months [range, 6-89 months]) (Table 3).

**Table 3:** Characteristics of the patients who underwent surgery

Surgery	Age (y)	Histology	T (cm)	N		M	TNM stage	RR	Initial response	Response at the end of follow-up (median 46.4 m, 6-80)	
TT	46.9	CPTC	0.9	0		0	I	Low	Excellent	NED	
HT	37.5	CPTC	1.1	1a		0	I	Low	Structural incomplete	NED	
TT	65.5*	CPTC	1.1	0		0	I	Low	Biochemical incomplete	NED	
TT	24.2	CPTC	1.5	0		0	I	Low	Excellent	NED	
TT	44.3	CPTC	0.8	1a		0	I	Low	Excellent	NED	
TT	32,3	CPTC	1.5	0		0	I	Low	Indeterminate	NED	
TT	40,4	CPTC	0,7	0		0	I	Low	Excellent	NED	
TT	55,5	CPTC	1,1	0		0	I	Low	Excellent	NED	
TT	26,6	CPTC	9	0		0	I	Low	Excellent	NED	
TT	40	FVPTC	1,5	0		0	I	Low	Excellent	NED	

TT: total thyroidectomy; HT: hemithyroidectomy; T: tumor; N: node involvement; M: distant metastasis; RR: risk of recurrence; CPTC: classical papillary thyroid carcinoma; FVPTC: follicular variant of papillary thyroid carcinoma; NED: no evidence of disease.

* Patient who presented a serum Tg doubling time of 6 months with a posterior PMC adjacent to the trachea.

## DISCUSSION

As a follow-up strategy, active surveillance has been initially proposed in patients with localized low-risk prostate cancer, with the intention of delaying active treatment until the tumor revealed a significant progression, thus avoiding the side effects of previously proposed treatments (26). This approach can also be used in patients with slow-growing tumors, such as thyroid cancer, as several authors have shown (12-21). According to the 2015 American Thyroid Association Guidelines (23) for the treatment and follow-up of patients with thyroid cancer, active surveillance can be considered an alternative to immediate surgery in various situations: i) in very low-risk papillary microcarcinomas with no clinical or radiographic evidence of histologically adverse features, such as extrathyroidal extension, lymphovascular invasion, or metastatic lymph node involvement; ii) when no aggressive cytological variants of thyroid cancer are present; iii) in patients at high surgical risk because of comorbid conditions; iv) in patients expected to have a relatively short remaining life span (e.g., serious cardiopulmonary disease, other malignancies, very advanced age); and v) inpatients with concurrent medical or surgical issues that need to be addressed prior to thyroid surgery (23,27). The literature supports this approach as a safe and effective alternative to immediate surgery in properly selected patients (14-21).

In our study, it was surprising that only 25% of patients with a diagnosis of a PTC candidate for active surveillance accepted this modality as an alternative. To our knowledge, no other studies address the difficulty of implementing active surveillance, but it seems it is currently not an option most patients consider, at least in our country (28,29).

To date, reliable molecular features that can differentiate the small number of patients who will clinically progress under active surveillance have not been yet defined (30). Therefore, additional studies are needed to identify the specific risk factors that lead patients to choose surgery instead of active surveillance and to define the systematic follow-up process to establish frequency of neck ultrasound examinations, optimal serum TSH cutoff, and the potential role of serum Tg during surveillance, among others. Additionally, significant complications of thyroid surgery could also lead patients to choose active surveillance (19,31-33). The rate of permanent recurrent laryngeal nerve paralysis and permanent hypoparathyroidism was reported as high as 10% and 7.1%, respectively. Together, they can reach up to 35% (32). Such surgical complications can be avoided if observation is chosen as the first line of management (19,27,31,33).

The adequate management of patients with PTC under active surveillance requires the availability of an experienced multidisciplinary management team and high-quality neck ultrasounds (14). The lack of accuracy in measurements of tumor size might affect treatment-decision making and final outcomes. In relation to tumor kinetics, our results are consistent with previous studies conducted in Japan and United States (14-21). Tumor diameter growth of 3 mm or more from baseline during the first years of active surveillance was observed in about 17% of the patients because we included patients with a retrospective follow-up (see before). Small PTCs up to 1.5 cm in diameter, even above the traditional 1-cm cutoff, showed a similarly low likelihood of growth, and a small percentage of patients experienced tumor regression (14). This last phenomenon might occur due to local immune mechanisms and/or necrosis, triggered by FNAB’s traumatic effect on the intratumoral cell population (14,20). On the other hand, none of our patients experienced loco-regional metastases, distant metastasis, or specific death during active surveillance (14-21). As in previous investigations, the patients in our cohort who experienced an increase in tumor size were under 60 years old, with a mean age of 45.86 ± 17.3 years (14,17,19). This subgroup of patients had TSH levels in the lower part of the reference range without hormone replacement therapy, not supporting the association theory regarding elevated serum TSH levels and increased tumor volume in the first 2 years of follow-up (34). Increased serum Tg levels were observed in 5% of patients. However, none of them showed an increase in tumor diameter. Until now, no conclusive studies had been conducted on the prognostic value of basal serum thyroglobulin levels in tumor progression during active surveillance (35). In our cohort, 23% of the patients decided to undergo surgery despite no increase in tumor size, mostly due to anxiety stemming from the follow-up. All of them were classified as low risk of recurrence with an initial excellent response to treatment and no evidence of disease after 4.6 years of follow-up.

In conclusion, this is the first study reported in Latin America related to active surveillance in appropriately selected patients with PTC. Only a quarter of patients who were offered to undergo an active surveillance of the PTC accepted this procedure. Additionally, around 10% of patients decided to abandon active surveillance due to anxiety. Although surgical treatment continues to be the standard procedure in most patients with thyroid carcinoma, this new approach would be safe, effective, and easily applicable in centers with extensive experience in the management of patients with thyroid cancer.
